# What Affects Sports Participation and Life Satisfaction Among Urban Residents? The Role of Self-Efficacy and Motivation

**DOI:** 10.3389/fpsyg.2022.884953

**Published:** 2022-04-28

**Authors:** Guo Yu, Yu Song

**Affiliations:** ^1^School of Economics and Management, Shanghai University of Sport, Shanghai, China; ^2^Institute of Sport Business, Loughborough University, London, United Kingdom

**Keywords:** urban residents, sports participation, sports participation motivation, self-efficacy, life satisfaction

## Abstract

As a result of the global pandemic, new awareness and perceptions of physical and mental health are changing. How to promote people's participation in sports to improve their physical fitness and how to increase their life satisfaction are urgent issues for attention. This study is aimed to discover the mechanisms that influence sports participation and life satisfaction among urban residents, focusing on the role of self-efficacy and motivation. A questionnaire survey on sports participation of Shanghai residents in China found that self-efficacy significantly and positively influenced sports participation; sports participation significantly and positively influenced life satisfaction; and motivation positively influenced sports participation and mediated between self-efficacy and sports participation, but sports participation did not mediate between self-efficacy and life satisfaction. Therefore, it is important to enhance people's self-confidence in sports participation and establish the concept of sports for all people, so as to strengthen people's motivation to participate in sports, and to guide people to exercise actively through national fitness, so that more people can be happy in sports.

## Introduction

In the past few years, sports participation has been a major research focus (Silva et al., 2020). Many studies have shown that the spread of epidemics can lead to reduced participation in physical activities (Ammar et al., 2021), increased incidence of mental illness (Ammar et al., 2020, 2021), and significantly reduced life satisfaction (Brooks et al., 2020). However, studies of sports participation and life satisfaction have focused on students (Chen et al., 2020; Wang et al., 2020; Turgut, 2021) and older adults (Kelly et al., 1987; Stathi et al., 2002; Heo et al., 2013), with a lack of research on groups that make up a much larger proportion of the population. Therefore, in the context of the epidemic becoming the norm, it is essential to study how sports and physical activities can promote life satisfaction and enhance mental health in a wider population (Ammar et al., 2020). According to social cognitive theory, human behavior is mainly determined by self-efficacy, and the motivation influenced by self-efficacy has an important impact on the results of behavior (Bandura, 1977). Self-efficacy refers to an individual's judgment of their own ability and confidence that their actions can achieve results. Self-efficacy produces motivation, which promotes behavior to form results (Pelletier et al., 1995). Some people have low self-efficacy in sports and are not confident about the results of participating in sports, which makes them lack in motivation to participate in sports (Lim, 2009). At the same time, in the research on the influence mechanism of sports participation, there are many studies on the influence relationship between variables, respectively (Ryckman and Hamel, 1993; Wang, 2003; Bowker, 2006; Richards, 2018), while there are few studies on the influence relationship between variables under the unified framework (Ha et al., 2017; Te Velde et al., 2018). Therefore, it is very necessary to explore the relationship between the influencing factors of sports participation under the unified framework.

Nowadays, more and more people are aware of the importance of physical and mental health in all aspects of economic, political, and social development (Darbaz et al., 2022). Although the government vigorously calls on people to actively participate in sports (Chen et al., 2019), many people have not formed the habit of regularly participating in physical exercise. In the 5 years from 2015 to 2020, the proportion of the sports population in has China increased by nearly 4 percentage points, accounting for about 37.2%. Although China's population participating in physical exercise is gradually increasing but when compared with other developed countries, more than 40% of the proportion, there is still a certain gap, is far from reaching the level of “full participation” (Jiang et al., 2019).

China's urban population accounted for 63.89% of its total population in 2021, and this proportion is expected to continue to increase, according to the National Bureau of Statistics (NBS), as the country's urbanization process deepens. Because urban residents are better than the rural population in terms of sports consumption ability, sports participation awareness, sports infrastructure, and sports activity organization, the residents of large cities, therefore, contribute to a greater extent to the achievement of fitness for all (Li et al., 2021). This research takes urban residents as the research object, discusses the influencing factors of urban residents' sports participation from the perspective of sports participants' self-ability perception, and further develops the research on influencing factors of sports participation. At the same time, this research also explored the relationship between sports participation and life satisfaction, which further enriched the research on the role of sports participation.

## Literature Background and Hypotheses

### Self-Efficacy and Sports Participation

Self-efficacy is a concept obtained by Bandura (1977) based on the analysis of the relationship between knowledge and knowledge acquisition. It refers to the judgment of one's own ability and the confidence level of one's ideal belief to achieve the expected results. The role of self-efficacy lies in influencing people's choice of behavior direction and the degree of persistence and effort in the choice and ultimately affects people's rational and emotional responses when facing things (Bandura, 1977). As one of the variables of social cognitive theory, self-efficacy has a significant impact on participation in sports behavior in the prediction (Dzewaltowski, 1989). The stronger self-efficacy will more actively be involved in the sport, high self-efficacy of sports participation has a considerable boost (Feltz et al., 2008), and the ability to inspire the enthusiasm of sports participation and interest, which to a certain extent, also can enhance the time and intensity of sports participation (Anderson-Butcher et al., 2011). Studies have shown that high self-efficacy is closely related to physical exercise (Sullum et al., 2000). People with a keen sense of self-efficacy believe that they can successfully complete related sports projects, and their sports participation process will be smoother (Bandura, 1977). Therefore, the following assumptions are proposed:

H1. The self-efficacy is positively related to the sports participation.

### Self-Efficacy and Sports Participation Motivation

Sports participation motivation is the product of internal and external influences, and its formation and development are formed from two aspects: one is the promotion of internal needs, the other is the induction of human emotional factors or social environment (Jiao and Qian, 2002). When the sports participation motivation is formed, individuals can maintain the state of exercise for a certain period (Zhang and Wang, 2019). Among the factors influencing sports participation, motivation is the most important among many comparative items (Ragheb and Tate, 1993). In the constrains-motivation-behavior relationship model proposed by Alexandris et al. (2002), the motivation and behavior of participating in sports change in the same direction. Stipek (1998) believes that motivation can drive and maintain athletes' participation in sports. The change of internal and external factors will directly affect the motivation and ultimately change the behavior results (Pelletier et al., 1995). Therefore, the motivation for sports participation cannot fully and effectively cause a change of the behavior results of participation (Zhang and Wang, 2019). According to the research results of Dogan (2017), self-efficacy provides motivation, which in turn affects the results of behavior. Therefore, it is necessary to link motivation with self-efficacy in the research on sports participation. Gao et al. (2008) showed in the research results that the stronger the sense of self-efficacy, the sports motivation will also increase. Lim et al. (2010) found in their research that there is a strong positive relationship between self-efficacy and motivation and sports participation among undergraduate students. Hutchins (2008) showed in the research results that there is a close relationship between self-efficacy and motivation in the physical exercise participation. Although the current research has explored the relationship between motivation and self-efficacy and the relationship between self-efficacy and sports participation, there is no research that puts them together to discuss the interaction between urban residents.

Self-efficacy reflects an individual's confidence and self-perception of the ability to perform a specific task and determines an individual's behavioral decision (Gibbs, 2009). Self-efficacy is a precursor factor to the level of individual potential motivation, which can directly determine an individual's behavioral motivation (Bandura, 1977). The expectancy-value theory of achievement motivation indicates that individual motivation is related to the beliefs of an individual in ability, the expectation of success, and subjective task value (Wigfield, 1994). However, self-efficacy belongs to the beliefs of an individual in his or her own ability and an expectation on whether he or she can accomplish something well, so self-efficacy has a direct impact on motivation (Bryant and Bates, 2015). People with high self-efficacy think they will do well in sports, so they are more motivated to participate in sports and they feel more strongly about participating in sports. Therefore, the following assumptions are proposed:

H2. The self-efficacy is positively related to the sports participation motivation.

### Motivation and Sports Participation

Caspersen et al. (1985) defined physical activity as any voluntary movement produced by skeletal muscles that results in increased energy expenditure. The cognitive, emotional, and action components of physical activity enable it to be viewed as a behavior (Hirvensalo and Lintunen, 2011). As a subclass of physical activity, sports participation is planned, organized, and usually aimed at providing pleasure to the participants (Palacios-Cena et al., 2012). Sports participation is measured by the frequency, intensity, and duration of exercise (Hirvensalo and Lintunen, 2011).

Individual participation attitude and motivation are important influencing factors for individual sports participation (Deci and Ryan, 1985). Shank (1999) research divided the factors that affect people's sports participation into three aspects: the first is individual factors, such as personal motivation, perception, attitude, and other psychological processes; the second is group factors, such as culture, social class, reference group, and family social culture; the third is environmental factors, such as venue environment, social environment, and sports atmosphere. In terms of individual awareness and attitude, the influence of sports participation motivation on sports participation behavior has been concerned by many studies (Telama et al., 1997). The influence of self-efficacy on sports participation has also been paid attention by researchers, who believe that self-efficacy has a significant impact on improving interest in sports participation (Richards, 2018; Ouyang et al., 2019).

According to self-determination theory, motivation is closely related to behavior, and the enhancement of motivation will lead to more positive behavior (Ryan and Deci, 2000). Motivation, as a state in the change and development of demand, is the fuse that causes behavior (Deci and Ryan, 1985). In the field of sports research, motivation can be the starting point for participation in sports (Gill et al., 1983). At the same time, no matter the type of motivation is intrinsic motivation or extrinsic motivation (Pelletier et al., 1995), it can be significantly positively correlated with physical exercise activity behaviors (Chen et al., 2006). Studies have shown that it is precise because of a high degree of motivation that exercise participants can continuously carry out exercise activities (Longhurst and Spink, 1987). Therefore, sports participation motivation can significantly affect sports participation and positively change with sports participation. Therefore, the following hypothesis is proposed:

H3. Sports participation motivation is positively related to the sports participation.

### The Mediating Effect of Motivation Between Self-Efficacy and Sports Participation

The higher people's self-efficacy, the more confident they are about completing the physical activity, and the higher their level of physical participation will be. In the core theory of self-efficacy, motivation is created by self-efficacy, and with the change of the strength of self-efficacy, it influences and promotes the change of behavior results (Bandura, 1977). A high sense of self-efficacy will have an impact on the degree of motivation (Narciss, 2004), and the relationship between self-efficacy and motivation is not easy to be changed by a different range of conditions (Bandura, 1993). Some research results show that motivation affects behavioral efficiency, and self-efficacy affects behavioral results (Niehaus et al., 2012), and with the continuous increase of self-efficacy, the motivation to participate in sports is also constantly enhanced (Marcus et al., 1998). Self-efficacy, motivation, and participating behavior are positively linked (Etinkalp and Türksoy, 2011; Fawcett et al., 2011). The higher the self-efficacy, the stronger the desire to achieve their goals through participation in sports, and the more likely they were to participate in sports. Therefore, self-efficacy can improve the level of sports participation by enhancing the motivation of sports participation. Therefore, the following hypothesis is proposed:

H4. Sports participation motivation plays a mediating role between self-efficacy and the sports participation.

### Sports Participation and Life Satisfaction

As a cognitive dimension, life satisfaction is the key to measure subjective wellbeing (Giacomoni, 2004). The definition of life satisfaction adopted in this study will be in the field of social research. Life satisfaction is a cognitive assessment of an individual's living conditions for most of the time or for a period of time-based on his or her standards in the overall sense and is an important parameter to measure the quality of life of people in a certain society (Diener et al., 1999; Schwartz and Strack, 1999). The results have shown that sports participation has a positive impact on subjective wellbeing and life satisfaction: Becchetti et al. (2008) verified that in the team and individual sports, both active and passive participants in sport activity positively improved their life satisfaction; Piko and Noemi Keresztes (2006) found that students who participated in physical exercise more actively had higher life satisfaction and better physical and mental health; Pawlowski et al. (2011) proved that although participating in sports at different ages would have different effects on subjective wellbeing, overall, sports participation was positively related to subjective wellbeing; Moradi et al. observed that moderate and frequent participation in sports is beneficial to increase life satisfaction; Ammar et al. (2020) concluded that participation in indoor and outdoor physical activities was effective in improving mental health. Therefore, the following hypothesis is proposed:

H5. Sports participation is positively related to life satisfaction.

### The Mediating Effect of Sports Participation Between Self-Efficacy and Life Satisfaction

People with a high sense of self-efficacy tend to be more confident in themselves and they fully believe that they can do certain tasks well and therefore they have a higher level of satisfaction in their lives (Machmud, 2018). Not only that, these people with high self-efficacy, who are willing to try different things and also better able to complete related tasks, have less frustration in their lives and therefore they are more satisfied with their life (Csikszentmihalyi and Wong, 2014).

In sports contexts, people with a higher self-efficacy believe that they are better able to perform all kinds of sports and therefore their sports participation is higher (Allison et al., 1999), resulting in better physical and psychological outcomes (Kelinske et al., 2001). At the same time, participants with a greater sense of self-efficacy are more likely to have positive feelings and feedback during exercise (McAuley and Jacobson, 1991), they feel happier and more fulfilled and therefore, have higher levels of mental health and life satisfaction. The impact of self-efficacy on life satisfaction can be mediated through sports participation. Therefore, the following hypothesis is proposed:

H6: Sports participation plays a mediating role between self-efficacy and life satisfaction.

### Hypothesis Model

**Figure 1:**
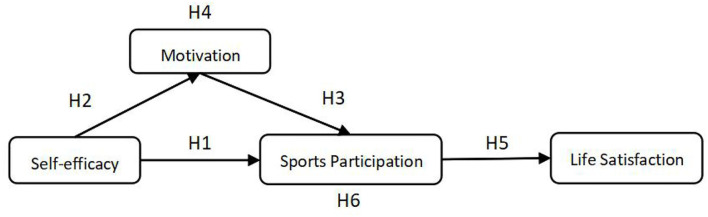
Conceptual framework and research hypotheses of the study.

To summarize, people's self-efficacy is a decisive factor in motivation and behavior to participate in sports (Feltz et al., 2008). An increased sense of self-efficacy motivates people to participate, and along with increased participation in exercise, people become physically and mentally healthy, ultimately increasing satisfaction (Lim, 2009). Sports participants, whether healthy or ill, perform better on this indicator of life satisfaction (Diaz et al., 2019). Motivation spurs people to participate in sport and remains for a period of time (Stiles, 1967; Frederick and Ryan, 1993; Downward et al., 2014). At the same time, self-efficacy positively influences motivation, with higher self-efficacy meaning stronger motivation (Jinks and Lorsbach, 2003). This study suggests that self-efficacy has positive effects on motivation and sports participation, motivation is positively related to sports participation, at the same time, motivation plays a mediating role in the relationship between self-efficacy and the sports participation, and the sports participation positively affects life satisfaction. Finally, sports participation mediates the relationship between self-efficacy and life satisfaction. The conceptual model is shown in Figure 1.

## Research Methods

### Sampling and Data Collection

To test the hypotheses, this research saw Shanghai residents as the research object and collected the data related to their sports participation by sending questionnaires to the residents in the main downtown areas of Shanghai—Huangpu District, Xuhui District, Changning District, Jing'an District, Putuo District, Hongkou District, Yangpu District, and Pudong New District. In this survey, a total of 670 questionnaires were sent out and 670 were received with a recovery rate of 100%, of which 645 were valid with an effective rate of 96% (Table 1). Among them, male residents (57.2%) are more than female residents (42.8%), 77.1% of the residents are in the 19–45 years range, 47% of the residents in the individual monthly income are in the range of U4,000RMB–U8,000RMB, and 39.7% of spectators had university or postgraduate education level. Moreover, the main occupations of respondents were general practice staff (16%), industrial worker (14.4%), student (13%), and businessperson (12.9%). The proportions of respondents in terms of gender, age, income, education, and occupation basically match the distribution of the seventh population census in Shanghai (Shanghai Municipal People's Government, 2021), indicating the representativeness of the sample. A small number of questions and answers for minors under the age of 18 are conducted with parental consent.

**Table 1:** Demographic characteristics of the sample (*N* = 645).

**Items**	**Percentage (%)**
**Gender**	
Female	57.2
Male	42.8
**Age**	
18 s and below	8.2
19–25 s	29.1
26–35 s	24.7
36–45 s	23.3
46–59 s	10.2
60 s and above	4.5
**Monthly income**	
RMB 2,000 and below	15.2
RMB 2,001–4,000	13.3
RMB 4,001–6,000	24.5
RMB 6,001–8,000	22.5
RMB 8,001–10,000	13.5
RMB 10,001 and above	11.0
**Education**	
High school and below	60.3
College degree	16.1
Bachelor degree	18.3
Postgraduate degree and above	5.3
**Occupation**	
Government employee	8.8
Professional and technical staff	11.8
General practice staff	16.0
Businessperson	13.0
Private business owner	12.9
Students	6.7
Industrial workers	14.4
Unemployed	1.7
Farmer	1.7
Retiree	4.2
Housewife or househusband	1.6
Others	7.3

### Variable Measures

In existing studies, the scale for measuring relevant variables is relatively mature. After collecting the scale, the project team combined it with the field of sports and made some modifications and adjustments to the specific items in the scale according to the suggestions given by experts in the field of sports and the results discussed by the research team, so as to better apply to this research. The study included four scales: sports participation, self-efficacy, life satisfaction, and sports participation motivation. All the items in the scale were measured on a 7-level Likert scale, with 1–7 points from “strongly disagree” to “strongly agree”.

Sports participation adopted the scale used by Robertson and Emerson (2010), which consists of three items: participating in sport regularly, participating in sport more often than people around me, and people around me think I participate in sport regularly. Self-efficacy referred to by Zhang and Schwarzer (1995) formulated by the Chinese general self-efficacy scale consists of seven items: I can overcome whatever happens, I believe in my own ability to face difficulties calmly, persistence allows me to overcome difficulties, to find solutions, to persevere in any situation, to persevere and achieve results, and to accomplish my goals even if others do not support me. Life satisfaction adopted the scale with four items compiled by Lucas et al. (1996): good living conditions, life currently close to ideal, satisfied with life, and getting what you want. The motivation scale referred to the simplified version of the exercise motivation scale constructed by Chen et al. (2013) that include five dimensions: fun, ability, appearance, health, and social interaction.

## Results

### Confirmatory Factor Analysis of the Model

In this study, the reliability and validity of the data were tested by confirmatory factor analysis, and AMOS24.0 software, as well as SPSS27.0 software were adopted. *X*^2^ test, root mean square error of approximation (RMSEA), root mean square residual (RMR), goodness of fit index (GFI), adjusted goodness-of-fit (AGFI), parsimony goodness-of-fit index (PGFI), Tucker-Lewis index (TLI) and comparative fit index (CFI) are important indicators of how well the model fits (Schermelleh-Engel et al., 2003). The results show that the model fits the data well (*X*^2^/*df* = 2.738, RMSEA = 0.052, RMR = 0.118, GFI = 0.921, AGFI = 0.889, PGFI = 0.655, TLI = 0.957, and CFI = 0.967). As shown in Table 2, the composite reliability of all structural variables is higher than the recommended level (0.60), and the average variance extracted (AVE) is also higher than the recommended level (0.50), indicating that the measurement of related structural variables has good reliability. The standardized factor load of structural variables was approximately higher than 0.5 and was significant at α = 0.01 level, which indicates higher validity of the scale.

**Table 2:** Loads, standard deviations, reliability estimates, and convergent validity estimates.

**Latent variables**	**Observed variables**	**Load**	**S.D**	**AVE**	**CR**	**Cronbach'α**
Sports participation	1. I often participate in sports	0.899	0.177	0.788	0.917	0.916
	2. I participate in sports more than the people around me	0.918				
	3. People around me think I often participate in physical exercise	0.845				
Self-efficacy	1. No matter what comes my way during exercise, I'm usually able to handle it.	0.731	0.133	0.614	0.864	0.865
	2. I can remain clam when facing difficulties because I can rely on my sports skills.	0.773				
	3. I can always manage to solve difficult problems in participating in sports if I try hard enough.	0.879				
	4. When I am confronted with a problem in participating in sports, I can usually find several solutions.	0.744				
	5. I am confident that I could deal efficiently with unexpected events during exercise.	0.827				
	6. It is easy for me to stick to my aims and accomplish my exercise goals.	0.839				
	7. If someone opposes me, I can still achieve my exercise goals.	0.749				
Life satisfaction	1. In most ways my life is close to my ideal.	0.796	0.119	0.646	0.927	0.932
	2. The conditions of my life are excellent.	0.778				
	3. I am satisfied with my life.	0.802				
	4. So far, I have gotten the important things I want in life.	0.833				
Motivation	1. To enhance the feelings and friendship with friends	0.757	0.083	0.483	0.928	0.939
	2. To meet new people	0.738				
	3. To maintain good social relationships	0.748				
	4. To maintain or improve your body shape	0.675				
	5. Weight control	0.600				
	6. Makes me more physically attractive	0.589				
	7. To improve my existing motor skills	0.728				
	8. To acquire new motor skills	0.769				
	9. To maintain current athletic skill level	0.650				
	10. Keep my body and mind healthy	0.628				
	11. Live a healthy life	0.668				
	12. Have more energy	0.736				
	13. Keep a good mood	0.729				
	14. Enjoy a happy life	0.683				

### Main Effects

**Figure 2:**
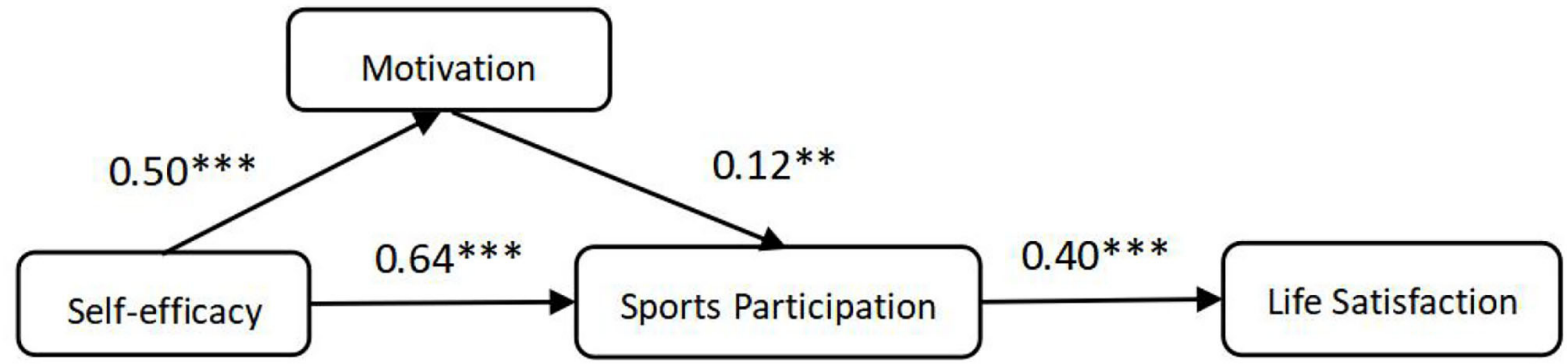
Relationship among sports participation, self-efficacy, and motivation. *p < 0.05; **p < 0.01; ***p < 0.001.

AMOS24.0 was used to test the main effect of the hypothetical model, and the results are shown in Figure 2. These statistics show that the structural model of the main effect fits well (*X*^2^/*df* = 2.342, RMSEA = 0.043, RMR = 0.034, GFI = 0.976, AGFI = 0.987, TLI = 0.988, and CFI = 0.967).

The results of the study showed that self-efficacy has significant positive effects on sports participation (γ = 0.64, *p* < 0.001). Self-efficacy has significant positive effects on sports participation motivations (γ = 0.50, *p* < 0.001). Sports participation motivations have significant positive effects on sports participation (γ = 0.12, *p* < 0.01). Sports participation has significant and positive effects on life satisfaction (γ = 0.40, *p* < 0.001). Therefore, H1, H2, H3, and H5 are supported.

### The Mediating Effect of Sports Participation Motivations

In this study, the 95% confidence interval (CI) was calculated by AMOS24.0 using the mediating effect of Bootstrapping test method on sports participation motivations. According to the results in Table 3, in the interaction between self-efficacy and sports participation with motivation as the mediating variable, the *Z*-values of the point estimation of the total effect, indirect effect, and direct effect of self-efficacy on sports participation are 21.707, 2.600, and 16.571, respectively, all of which were higher than 1.96. At 95% confidence level, neither bias-corrected Percentile Method nor Percentile Method includes 0 for direct and indirect effects. This indicates that motivations have a significant and partial mediating effect between self-efficacy and sports participation. This shows that self-efficacy can not only directly affect sports participation but also can indirectly affect sports participation by influencing the sports participation motivations, supporting H4.

**Table 3:** Results of the mediating effect of sports participation motivations.

**Variable**	**Point estimation**	**Product of coefficients**		**Bootstrapping**			
				**Bias-corrected 95% CI**		**Percentile 95% CI**	
		**SE**	***Z***	**Lower**	**Upper**	**Lower**	**Upper**
**Total effects**							
Self-efficacy → Sports participation	0.890	0.041	21.707	0.816	0.978	0.814	0.972
**Indirect effects**							
Self-efficacy → Sports participation	0.078	0.030	2.600	0.022	0.143	0.023	0.144
**Direct effects**							
Self-efficacy → Sports participation	0.812	0.049	16.571	0.722	0.914	0.716	0.910

### The Mediating Effect of Sports Participation

The results from Table 4 show that in the interrelationship between self-efficacy and life satisfaction with sports participation as the mediating variable, the Z values for the point estimates of the total and direct effects of self-efficacy on life satisfaction are 10.647 and 7.985, respectively, which are >1.96, and the indirect effect is 0.545, which is <1.96, and the indirect effect is not significant at the 95% confidence level of Bias-Corrected Percentile Method and Percentile Method included 0, the indirect effect was not significant, indicating that sports participation does not have a mediating effect in the relationship between self-efficacy and life satisfaction. H6 was not supported.

**Table 4:** Results of the mediating effect of sports participation.

**Variable**	**Point estimation**	**Product of coefficients**		**Bootstrapping**			
				**Bias-corrected 95% CI**		**Percentile 95% CI**	
		**SE**	***Z***	**Lower**	**Upper**	**Lower**	**Upper**
**Total effects**							
Self-efficacy → Life satisfaction	0.543	0.051	10.647	0.445	0.642	0.446	0.644
**Indirect effects**							
Self-efficacy → Life satisfaction	0.024	0.044	0.545	−0.065	0.109	−0.062	0.112
**Direct effects**							
Self-efficacy → Life satisfaction	0.519	0.065	7.985	0.392	0.648	0.393	0.649

## Research Conclusion and Implications

### Research Findings

This study explored the relationship among self-efficacy, motivations, sports participation, and life satisfaction through a questionnaire survey of Shanghai residents. The relevant conclusions of this study are as follows.

First, self-efficacy has a significant positive impact on sports participation. People with a keen sense of self-efficacy are more confident, they tend to think that they can better complete all kinds of sports-related movements, so they can more easily participate in any kind of sports (Lu, 2021). Those with a weak sense of self-efficacy are often less willing to participate in sports because they are concerned that they cannot well complete and master the relevant skills of sports (Yan and Li, 2019). Therefore, self-efficacy has an impact on individuals' sports participation behavior from the perspective of their self-perception.

Second, sports participation has a significant positive impact on life satisfaction. Sports can bring many benefits to participants, such as better body shape (Dyremyhr et al., 2015), better physical health (Pagan, 2019), increased mental optimism (Varca et al., 2010), and higher work efficiency. All of these have a significant effect on people's life satisfaction (Poulsen et al., 2008).

Thirdly, self-efficacy has a significant positive effect on sports participation motivations. People with high self-efficacy believe that they can complete sports activities well and that their motivations (such as appearance, health, and social interaction) can be better realized through participating in sports (Pelletier et al., 1995, 2013). Therefore, people with high self-efficacy are more motivated to participate in physical activity.

Fourth, sports participation motivations can significantly positively affect sports participation. The more motivated the persons are to participate in sports, the more eager they are to exercise to achieve weight loss, health, and social goals (Mota and Esculcas, 2002; Silva et al., 2011; Stefanović, 2013). In this way, they are more likely to take action to get involved in sports and thus achieve these sports participation goals for themselves (Nicholls et al., 2015). Therefore, the more motivated people are, the higher their levels of participation in sports are.

Fifth, sports participation motivations play a mediating role between self-efficacy and sports participation. Self-efficacy is high, they think they can better complete sports, sports participation goals can be better achieved (Duda, 1995). Therefore, their motivation for sports participation will become stronger due to the high level of self-efficacy, which leads them to actively participate in sports, to realize their motivation for sports participation.

Finally, there was no mediating effect of sports participation between self-efficacy and life satisfaction. This is similar to the findings of the studies by Han (2015) and Man (2017). The region selected for the sample has a good level of sports development, participation in sport is more common (Xiao et al., 2020) and people internalize sport as a lifestyle or health philosophy. Shanghai also has a high level of economic development and the second-highest average wage in China (Textor, 2021), with higher income levels allowing people to improve their life satisfaction in more alternative ways (Inoue et al., 2017).

### Practical Implications

The results of this study provide some implications for the government and social organizations to establish the awareness of sports participation for all and improve the level of residents' sports participation and people's life satisfaction.

The first implication is to enhance people's confidence in sports participation which can help to establish the concept of nationwide sports participation. The government and social organizations should vigorously promote the belief that everyone can participate in sports and enhance the confidence of all people in sports participation. The social atmosphere should convey the idea that men and women, regardless of their age and physical condition, can find sports that suit them and participate in them. Especially for some special groups, such as patients with certain diseases, older people, physically weak people, and pregnant women, they often think that they are not suitable to participate in sports or cannot participate in sports well. However, this view is not true, because these groups as long as they find the right sports and participate in them, it is extremely beneficial to their physical health recovery (Robertson and Emerson, 2010; Pagan, 2019). Therefore, the government and social organizations should publicize and educate the people, establish their confidence in sports participation, and let them join in sports with better cognition and more positive mental state. Movement function is not very developed for groups, such as obesity group, the group of women, their sports ability is relatively poor, but they still need to be set up by the government and the social organizations to the concept of sports participation, enhance their sports participation in confidence, as much as possible, so that they can participate in sports, to give them the benefits of fully enjoy sports.

Second, government and social organizations promote the benefits of sports and strengthen people's motivation to participate in sports. From the perspective of the government, it is necessary to pay attention to the publicity, formulate relevant policies to guide, and regulate the role of media in the publicity of sports, so that people can better receive positive information about participating in sports, realize the benefits of sports participation, and bring satisfaction and positive attitude to life (Kong et al., 2008). For example, in 2012, the General Administration of Sport of China issued the Guidance on the Administration of Microblog (We-Media) of the General Administration of Sport of China, which specifically elaborated the management of the content and development direction of media publicity, promoted the effectiveness of sports publicity, and inspired more people to join in sports. From the perspective of society, sports organizations should not only play a significant role in publicity, but also play the coverage role of individual communication, expand the scope of communication, so as to make the benefits brought by sports deeply rooted in people's hearts, improve the motivation intensity of people's sports participation, and ultimately improve their sports participation level.

Lastly, government and social organizations can guide exercise for happiness through national fitness so as to improve people's satisfaction with life. Without universal health, there can be no universal prosperity. National fitness is one of the effective ways to achieve national health and is an important method to improve people's life happiness. The government and social organizations need to create a pleasant sports atmosphere around us and improve people's life satisfaction through the effective development of national fitness activities. For example, the Norwegian sports system has been established to promote deeper and more enjoyable participation in sports by specific groups (e.g., women, children, the elderly, and the disabled), thus providing lower barriers to participation and greater opportunities (Seippel and Belbo, 2021), which has resulted in more than 90% of young people already joining sports clubs (Bakken, 2017) and contribute to the goal of “sport-for-all”. At the same time, individual sports participation to be actively involved in the exercise, feel the sports can bring benefits to the participants, including a more muscular body and a healthy body (Groot et al., 2020), better social relationships (Weiss et al., 1996), more optimistic state of mind (Feng et al., 2007), etc., so as to improve people's life satisfaction and happiness.

### Limitations and Prospects

Through the investigation of sports participants in Shanghai, this article studies the influence mechanism of self-efficacy and life satisfaction on sports participation under the mediating effect of motivations. This study has some limitations, which also point out the direction for future research. First, as Shanghai is one of China's mega-cities, the study of sports participants in Shanghai mainly tends to study urban sports participation. However, there are differences in the sports participation behavior of people in small- and medium-sized cities and rural areas (Li et al., 2021), and more research is needed to verify whether they are influenced by the same mechanisms in the future. Second, the influencing factors of sports participation are various (Downward et al., 2011; Wheeler, 2012; Timperio et al., 2013; Matta et al., 2021). However, this study only studied from the perspective of self-efficacy and motivations, and the effects of other factors need to be verified by more studies in the future. Thirdly, this study did not discuss the influence mechanism of different groups' sports participation (Biddle, 1995; Trost et al., 2002; Pauline, 2013). Therefore, whether the influencing mechanism of different group sports participation is the same, more studies are needed to test in the future.

## Data Availability Statement

The raw data supporting the conclusions of this article will be made available by the authors, without undue reservation.

## Ethics Statement

Ethical review and approval was not required for the study on human participants in accordance with the local legislation and institutional requirements. Written informed consent from the participants' legal guardian/next of kin was not required to participate in this study in accordance with the national legislation and the institutional requirements.

## Author Contributions

Both authors contributed to the data collection and analysis of the article, the control of the overall idea, the presentation of the final draft, and agreed to approve the final draft.

## Funding

This work was supported by the Chinese National Funding of Social Sciences (Grant Number 16CTY008).

## Conflict of Interest

The authors declare that the research was conducted in the absence of any commercial or financial relationships that could be construed as a potential conflict of interest.

## Publisher's Note

All claims expressed in this article are solely those of the authors and do not necessarily represent those of their affiliated organizations, or those of the publisher, the editors and the reviewers. Any product that may be evaluated in this article, or claim that may be made by its manufacturer, is not guaranteed or endorsed by the publisher.

## References

[B1] Alexandris K. Tsorbatzoudis C. Grouios G. (2002). Perceived constraints on recreational sport participation: investigating their relationship with intrinsic motivation, extrinsic motivation and amotivation. J. Leisure Res. 34, 233–252. 10.1080/00222216.2002.11949970

[B2] Allison K. R. Dwyer J. J. Makin S. (1999). Self-efficacy and participation in vigorous physical activity by high school students. Health Educ. Behav. 26, 12–24. 10.1177/109019819902600103 9952049

[B3] Ammar A. Mueller P. Trabelsi K. Chtourou H. Boukhris O. Masmoudi L.. (2020). Psychological consequences of COVID-19 home confinement: the ECLB-COVID19 multicenter study. PLoS ONE 15, e0240204. 10.1371/journal.pone.0240204 33152030 PMC7643949

[B4] Ammar A. Trabelsi K. Brach M. Chtourou H. Boukhris O. Masmoudi L.. (2021). Effects of home confinement on mental health and lifestyle behaviours during the COVID-19 outbreak: insights from the ECLB-COVID19 multicentre study. Biol. Sport 38, 9. 10.5114/biolsport.2020.96857 33795912 PMC7996377

[B5] Anderson-Butcher D. Riley A. Iachini A. Wade-Mdivanian R. Davis J. (2011). “Sports and youth development,” in Encyclopedia of Adolescence, eds R. J. R. Levesque (New York, NY: Springer).

[B6] Bakken A.. (2017). “Sosiale forskjeller i ungdomsidretten - fattigdomsproblem elle sosial gradient?” in Oppvekstrapporten, ed. Bufdir (Oslo: BUFDIR), 148–167.

[B7] Bandura A.. (1977). Self-efficacy: toward a unifying theory of behavioral change. Psychol. Rev. 84, 191–215. 10.1037/0033-295X.84.2.191 847061

[B8] Bandura A.. (1993). Perceived self-efficacy in cognitive development and functioning. Educ. Psychol. 28, 117–148. 10.1207/s15326985ep2802_3

[B9] Becchetti L. Pelloni A. Rossetti F. (2008). Relational goods, sociability, and happiness. Kyklos 61, 343–363. 10.1111/j.1467-6435.2008.00405.x

[B10] Biddle S. J. H.. (1995). “Exercise motivation across the life span,” in European Perspectives on Exercise and Sport Psychology. ed. S. J. H. Biddle (Champaign, IL: Human Kinetics Publishers), 3–25.

[B11] Bowker A.. (2006). The relationship between sports participation and self-esteem during early adolescence. Can. J. Behav. Sci. 38, 214–229. 10.1037/cjbs2006009

[B12] Brooks S. K. Webster R. K. Smith L. E. Woodland L. Wessely S. Greenberg N.. (2020). The psychological impact of quarantine and how to reduce it: rapid review of the evidence. Lancet 395, 912–920. 10.1016/S0140-6736(20)30460-8 32112714 PMC7158942

[B13] Bryant J. Bates A.J. (2015). Creating a constructivist online instructional environment. Tech. Trends 59, 71–22. 10.1007/s11528-015-0834-1

[B14] Caspersen C. J. Powell K. E. Christenson G. M. (1985). Physical activity, exercise, and physical fitness: definitions and distinctions for health-related research. Public Health Rep. 100, 126–131. 3920711 PMC1424733

[B15] Chen P Li F Harmer P. (2019). Healthy China 2030: moving from blueprint to action with a new focus on public health. Lancet Public Health. 4, e447. 10.1016/S2468-2667(19)30160-4 31493840

[B16] Chen S. Ho W. K. Y. Ahmed M. D. (2020). Physical activity and its relationship with life satisfaction among middle school students: a cross-culture study. Sustainability 12, 6932. 10.3390/su12176932

[B17] Chen S. P. Wang Y. B. Rong J. Z. Pan X. G. Bao J. (2013). The simplified version of the MPAM-R: reliability and validity. J. Beijing Sport Univ. 36, 66–78. 10.19582/j.cnki.11-3785/g8.2013.02.013

[B18] Chen S. P. Yan Z. L. Tan H. Y. (2006). Analysis on reliability and validity of MPAM-R in Chinese version. China Sport Sci. Technol. 42, 52–54. 10.16470/j.csst.2006.02.014

[B19] Csikszentmihalyi M. Wong M. M. H. (2014). “The situational and personal correlates of happiness: a cross-national comparison,” in Flow and the Foundations of Positive Psychology, eds F. Strack, M. Argyle, and N. Schwartz (Dordrecht: Springer), 69–88.

[B20] Darbaz I. Morris G. Tüzmen S. (2022). “Prevention and control strategies for the COVID-19 pandemic,” in COVID-19: From Bench to Bedside (Chapter 4) eds Barh D. Lundstrom K. (New York, NY: CRC Press).

[B21] Deci E. L. Ryan R. M. (1985). Intrinsic motivation and self-determination in human behavior. Contemp. Sociol. 3, 253. 10.1007/978-1-4899-2271-7

[B22] Diaz R. Miller E. K. Kraus E. Fredericson M. (2019). Impact of adaptive sports participation on quality of life. Sports Med. Arthrosc. Rev. 27, 73–82. 10.1097/JSA.0000000000000242 31046012

[B23] Diener E. Suh E. M. Lucas R. E. Smith H. L. (1999). Subjective well-being: three decades of progress. Psychol. Bull. 125, 276–302. 10.1037/0033-2909.125.2.276

[B24] Dogan U.. (2017). Student engagement, academic self-efficacy, and academic motivation as predictors of academic performance. Anthropologist 20, 553–561. 10.1080/09720073.2015.11891759

[B25] Downward P. Lera-Lopez F. Rasciute S. (2011). The zero-inflated ordered probit approach to modelling sports participation. Econ. Model. 28, 2469–2477. 10.1016/j.econmod.2011.06.024

[B26] Downward P. Lera-Lopez F. Rasciute S. (2014). The correlates of sports participation in Europe. Eur. J. Sport Sci. 14, 592–602. 10.1080/17461391.2014.880191 24498937

[B27] Duda J. L.. (1995). “Motivation in sport settings: a goal perspective approach.” in Motivation in Sport and Exercise, ed. G. C. Roberts (Champaign, IL: Human Kinetics Books), 57–91.

[B28] Dyremyhr A. E. Diaz E. Meland E. (2015). How adolescent subjective health and satisfaction with weight and body shape are related to participation in sports. J. Environ. Public Health 2014, 1–7. 10.1155/2014/851932 25013414 PMC4074947

[B29] Dzewaltowski D. A.. (1989). Toward a model of exercise motivation. J. Sport Exerc. Psychol. 11, 251–269. 10.1123/jsep.11.3.251

[B30] Etinkalp Z. K. Türksoy A. (2011). Goal orientations and self-efficacy: as predictors of participation motivation in adolescent male soccer players. Soc. Behav. Person. Int. J. 39, 925–934. 10.2224/sbp.2011.39.7.925

[B31] Fawcett L. M. Garton A. F. Dandy J. (2011). Role of motivation, self-efficacy and parent support in adolescent structured leisure activity participation. Austral. J. Psychol. 61, 175–182. 10.1080/00049530802326792

[B32] Feltz D. L. Short S. E. Sullivan P. J. (2008). Self-efficacy in sport. Hum. Kinet. Champaign, IL. 10.5040/9781718206625

[B33] Feng L. U. Liu Y. Chen Q. (2007). Research on mental effect of citizens from four cities participating in sports activity in southwest region. China Sport Sci. Technol. 43, 15–22. 10.16470/j.csst.2007.04.003

[B34] Frederick C. M. Ryan R. M. (1993). Differences in motivation for sport and exercise and their relations with participation and mental health. J. Sport Behav. 16, 124–147. 27488255

[B35] Gao Z. Lee A. M. Harrison L. (2008). Understanding students' motivation in sport and physical education: from the expectancy-value model and self-efficacy theory perspectives. Quest 60, 236–254. 10.1080/00336297.2008.10483579

[B36] Giacomoni C. H.. (2004). Bem-estar subjetivo: em busca da qualidade de vida. Temas em: Psicologia da SBP. 12, 43–50.

[B37] Gibbs S. R.. (2009). Exploring the influence of task-specific self-efficacy on opportunity recognition perceptions and behaviors. Front. Entrepren. Res. 29, 1–15. Available online at: http://digitalknowledge.babson.edu/fer/vol29/iss6/1

[B38] Gill D. L. Gross J. B. Huddleston S. (1983). Participation motivation in youth sports. Int. J. Sport Psychol. 14, 1–14.

[B39] Groot S. D. Kouwijzer I. Valent L. Hagoort M. Post M. (2020). Sport participation after the handbikebattle: benefits, barriers, facilitators from the event–a follow-up survey. Spinal Cord Series Cases 6, 54. 10.1038/s41394-020-0301-x 32601299 PMC7324566

[B40] Ha S. R. Cho J.-H. Yoon Y.-K. (2017). Moderating effects of sport-event participation experience through the relationships among exercise motivation, physical self-efficacy, and sociality improvement in youth sports club members. Kor. J. Phys. Educ. 56, 173–183. 10.23949/kjpe.2017.09.56.5.14

[B41] Han G. S.. (2015). The relationship between self-efficacy and college life satisfaction: moderating effect of leisure sport participation level. J. Korea Acad. Indust. Cooper. Soc. 16, 2478–2485. 10.5762/KAIS.2015.16.4.2478

[B42] Heo J. Stebbins R. A. Kim J. Lee I. (2013). Serious leisure, life satisfaction, and health of older adults. Leisure Sci. 35, 16–32. 10.1080/01490400.2013.739871

[B43] Hirvensalo M. Lintunen T. (2011). Life-course perspective for physical activity and sports participation. Eur. Rev. Aging Phys. Activity 8, 13–22. 10.1007/s11556-010-0076-3

[B44] Hutchins M. D.. (2008). Relationships among self-efficacy, self-motivation, and other factors affecting physical activity: health implications for health education (PhD thesis). Southern Illinois University Carbondale, Carbondale, IL, United States.

[B45] Inoue Y. Sato M. Filo K. Du J. Funk D. C. (2017). Sport spectatorship and life satisfaction: a multicountry investigation. J. Sport Manag. 31, 419–432. 10.1123/jsm.2016-0295

[B46] Jiang Z. M. Zhu C. G Wang S. Y. Cao S. D. (2019). Comparative analysis of the focus of sports population research at home and abroad. J. Nanjing Sports Instit. 2, 18–23. 10.15877/j.cnki.nsin.2019.03.004

[B47] Jiao Y. F. Qian Z. M. (2002). To explore the effects of phychologocal factors upn the motivation in physical exercise: the application of one kind of multiple motivations. J. Nanjing Sports Instit. 16, 68–70. 10.15877/j.cnki.nsic.2002.05.032

[B48] Jinks J. Lorsbach A. (2003). Introduction: motivation and self-efficacy belief. Read. Writing Quart. 19, 113–118. 10.1080/10573560308218

[B49] Kelinske B. Mayer B. W. Chen K. (2001). Perceived benefits from participation in sports: a gender study. Women Manag. Rev. 16, 75–84. 10.1108/09649420110386601

[B50] Kelly J. R. Steinkamp M. W. Kelly J. R. (1987). Later-life satisfaction: does leisure contribute? Leisure Sci. 9, 189–199. 10.1080/01490408709512159

[B51] Kong H. S. Lee K. S. Lee S. Y. Yu J. H. Hong A. (2008). The comparison of health status and satisfaction with life according to paticipation in exercise program for the elderly. J. Korean Soc. Health Educ. Promot. 25, 45–57.

[B52] Li C. Chen J. H. Liu X. H. Ren S. Q. (2021). Can physical exercise improve the residents' health? Front. Public Health 9, e707292. 10.3389/fpubh.2021.707292 34268293 PMC8275878

[B53] Lim K. C. Khor P. H. Tham Y. C. (2010). Relationship among attitude, self-efficacy, motivation and leisure-time physical activities participation of undergraduate students. J. Sports Sci. Technol. 10, 249–252.

[B54] Lim K. C.. (2009). University students' attitude, self-efficacy and motivation regarding leisure time physical participation. J. Pendidik Dan Pendidikan. 24, 1–15. 12416842

[B55] Longhurst K. Spink K. S. (1987). Participation motivation of australian children in-volved in organized sport. Can. J. Sport Sci. 12, 24. 3594314

[B56] Lu J. H.. (2021). Self-efficacy, competition outcome, and causal attributions in sport (PhD thesis). University of North Carolina at Greensboro, Greensboro, NC, United States.

[B57] Lucas R. E. Diener E. Suh E. (1996). Discriminant validity of well-being measures. J. Pers. Soc. Psychol. 71, 616–628. 10.1037/0022-3514.71.3.616 8831165

[B58] Machmud S.. (2018). The influence of self-efficacy on satisfaction and work-related performance. Int. J. Manag. Sci. Business Admin. 4, 43–47. 10.18775/ijmsba.1849-5664-5419.2014.44.1005

[B59] Man J. H.. (2017). Relationship between Chinese residents' social recognition and self-perceived health–mediating effects of sports participation. J. Wuhan Instit. Phys. Educ. 51, 95–100. 10.15930/j.cnki.wtxb.2017.09.016

[B60] Marcus B. H. Bock B. C. Pinto B. M. Forsyth L. Roberts M. B. Traficante R. M. (1998). Efficacy of an individualized, motivationally-tailored physical activity intervention. Ann. Behav. Med. 20, 174–180. 10.1007/BF02884958 9989324

[B61] Matta P. N. Baul T. D. Loubeau K. Sikov J. Spencer A. E. (2021). Low sports participation is associated with withdrawn and depressed symptoms in urban, school-age children. J. Affect. Disord. 280, 24–29. 10.1016/j.jad.2020.11.076 33221604 PMC7736521

[B62] McAuley E. Jacobson L. (1991). Self-efficacy and exercise participation in sedentary adult females. Am. J. Health Promot. 5, 185–207. 10.4278/0890-1171-5.3.185 10146835

[B63] Mota J. Esculcas C. (2002). Leisure-time physical activity behavior: structured and unstructured choices according to sex, age, and level of physical activity. Int. J. Behav. Med. 9, 111–121. 10.1207/S15327558IJBM0902_03 12174530

[B64] Narciss S.. (2004). The impact of informative tutoring feedback and self-efficacy on motivation and achievement in concept learning. Exp. Psychol. 51, 214. 10.1027/1618-3169.51.3.214 15267129

[B65] Nicholls A. R. Perry J. L. Jones L. Sanctuary C. Clough P. J. (2015). The mediating role of mental toughness in sport. J. Sports Med. Phys. Fit. 55, 824–834. Available online at: https://www.minervamedica.it/en/journals/sports-med-physical-fitness/article.php?cod=R40Y2015N07A0824 26360967

[B66] Niehaus K. Rudasill K. M. Adelson J. L. (2012). Self-efficacy, intrinsic motivation, and academic outcomes among latino middle school students participating in an after-school program. Hispanic J. Behav. Sci. 34, 118–136. 10.1177/0739986311424275

[B67] Ouyang Y. Wang K. Zhang T. Peng L. Song G. Luo J. (2019). The influence of sports participation on body image, self-efficacy, and self-esteem in college students. Front. Psychol. 10, 3039–3039. 10.3389/fpsyg.2019.03039 32116869 PMC7012809

[B68] Pagan R.. (2019). Sport participation, life satisfaction and domains of satisfaction among people with disabilities. Appl. Res. Qual. Life. 2020, 893–911. 10.1007/s11482-019-9711-y30268805

[B69] Palacios-Cena D. Fernandez-De-Las-Penas C. Hernandez-Barrera V. Jimenez-Garcia R. Alonso-Blanco C. Carrasco-Garrido P. (2012). Sports participation increased in spain: a population-based time trend study of 21 381 adults in the years 2000, 2005 and 2010. Br. J. Sports Med. 46, 1137–1139. 10.1136/bjsports-2012-091076 22685123

[B70] Pauline J. S.. (2013). Physical activity behaviors, motivation, and self-efficacy among college students. Coll. Stud. J. 47, 64–74.

[B71] Pawlowski T. Downward P. Rasciute S. (2011). Subjective well-being in european countries—on the age-specific impact of physical activity. Eur. Rev. Aging Phys. Activity 8, 93–102. 10.1007/s11556-011-0085-x

[B72] Pelletier L. G. Rocchi M. A. Vallerand R. J. Deci E. L. Ryan R. M. (2013). Validation of the revised sport motivation scale (sms-ii). Psychol. Sport Exerc. 14, 329–341. 10.1016/j.psychsport.2012.12.002

[B73] Pelletier L. G. Tuson K. M. Fortier M. S. Vallerand R. J. Briere N. M. Blais M. R. (1995). Toward a new measure of intrinsic motivation, extrinsic motivation, and amotivation in sports: the sport motivation scale (SMS). J. Sport Exerc. Psychol. 17, 35–53. 10.1123/jsep.17.1.35

[B74] Piko B. F. Noemi Keresztes M. A. (2006). Physical activity, psychosocial health, and life goals among youth. J. Commun. Health 31, 136. 10.1007/s10900-005-9004-2 16737174

[B75] Poulsen A. A. Ziviani J. M. Johnson H. Cuskelly M. (2008). Loneliness and life satisfaction of boys with developmental coordination disorder: the impact of leisure participation and perceived freedom in leisure. Hum. Move. Sci. 27, 325–343. 10.1016/j.humov.2008.02.004 18353475

[B76] Ragheb M. G. Tate R. L. (1993). A behavioural model of leisure participation, based on leisure attitude, motivation and satisfaction. Leisure Stud. 12, 61–70. 10.1080/02614369300390051

[B77] Richards A.. (2018). Kentuckian middle school students' self-efficacy and their participation in physical sports: a correlation study (PhD thesis). Liberty Universit, Lynchburg, VA. United States.

[B78] Robertson J. Emerson E. (2010). Participation in sports by people with intellectual disabilities in england: a brief report. J. Appl. Res. Intellect. Disabil. 23, 616–622. 10.1111/j.1468-3148.2009.00540.x

[B79] Ryan R. M. Deci E. L. (2000). Self-determination theory and the facilitation of intrinsic motivalion, social development, and well- being. Am. Psychol. 55, 68–78. 10.1037/0003-066X.55.1.68 11392867

[B80] Ryckman R. M. Hamel J. (1993). Perceived physical ability differences in the sport participation motives of young athletes. Int. J. Sport Psychol. 24, 270–283.

[B81] Schermelleh-Engel K. Moosbrugger H. Müller H. (2003). Evaluating the fit of structural equation models: Tests of significance and descriptive goodness-of-fit measures. Methods Psychol. Res. Online. 8, 23–74. Availalble online at: http://citeseerx.ist.psu.edu/viewdoc/download?doi=10.1.1.509.4258&rep=rep1&type=pdf

[B82] Schwartz N. Strack F. (1999). “Reports of subjective well-being: Judgmental processes and their methodological implications.” in Well-Being: The Foundations of Hedonic Psychology, ed. by D. Kahneman, E. Diener, and N. Schwartz (New York, NY: Russel Sage), 61–84.

[B83] Seippel R. Belbo J. S. (2021). Sport clubs, policy networks, and local politics. Int. J. Sport Policy 3, 1–21. 10.1080/19406940.2021.1898441

[B84] Shanghai Municipal People's Government (2021). Shanghai.gov.cn. Available online at: http://service.shanghai.gov.cn/sheninfo/newsdetail.aspx?Id=f10a9087-d795-4f49-81b0-eaea7fb3eee9 (accessed February 15, 2022).

[B85] Shank M. D.. (1999). Sports marketing: a strategic perspective. Medical Education. 8, 389–392. 10.1123/IJSC.2015-0051

[B86] Silva A. Monteiro D. Sobreiro P. (2020). Effects of sports participation and the perceived value of elite sport on subjective well-being. Sport Soc. 23, 1202–1216. 10.1080/17430437.2019.1613376

[B87] Silva M. N. Markland D. Carraça E. V. Vieira P. N. Coutinho S. R. Minderico C. S.. (2011). Exercise autonomous motivation predicts 3-yr weight loss in women. Med. Sci. Sports Exerc. 43, 728–737. 10.1249/MSS.0b013e3181f3818f 20689448

[B88] Stathi A. Fox K. R. McKenna J. (2002). Physical activity and dimensions of subjective well-being in older adults. J. Aging Phys. Activity 10, 76–92. 10.1123/japa.10.1.76 18048943

[B89] Stefanović R.. (2013). Influence of athletic recreational activities on health of workers in engineering industry. Res. Kinesiol, 41, 215–218.

[B90] Stiles M. H.. (1967). Motivation for sports participation in the community. Can. Med. Assoc. J. 96, 889–894. PMC1936158 6020893

[B91] Stipek D. J.. (1998). Motivation to learn: from theory to practice. Foreign Lang. Ann. 28, 116–120.

[B92] Sullum J. Clark B. M. King T. K. (2000). Predictors of exercise relapse in a college population. J. Am. College Health. 48, 175–180. 10.1080/07448480009595693 10650735

[B93] Te Velde S. J. Lankhorst K. Zwinkels M. Verschuren O. Takken T. De Groot J. (2018). Associations of sport participation with self-perception, exercise self-efficacy and quality of life among children and adolescents with a physical disability or chronic disease—a cross-sectional study. Sports Med. Open. 4, 1–11. 10.1186/s40798-018-0152-1 30112621 PMC6093823

[B94] Telama R. Yang X. Laakso L. Viikari J. (1997). Physical activity in childhood and adolescence as predictor of physical activity in young adulthood. Am. J. Prevent. Med. 13, 317–323. 10.1016/S0749-3797(18)30182-X 9236971

[B95] Textor C.. (2021). Average Annual Salary of an Employee in China 2020, by Region. Statista. Available online at: https://www.statista.com/statistics/278350/average-annual-salary-of-an-employee-in-china-by-region/ (accessed 24 February, 2022).

[B96] Timperio A. F. van Stralen M. M. Brug J. Bere E. Chinapaw M. J. De Bourdeaudhuij I.. (2013). Direct and indirect associations between the family physical activity environment and sports participation among 10-12 year-old european children: testing the enrg framework in the energy project. Int. J. Behav. Nutr. Phys. Activity 10, 1–10. 10.1186/1479-5868-10-15 23374374 PMC3621808

[B97] Trost S. G. Pate R. R. Sallis J. F. Freedson P. S. Taylor W. C. Dowda M.. (2002). Age and gender differences in objectively measured physical activity in youth. Med. Sci. Sports Exerc. 34, 350–355. 10.1097/00005768-200202000-00025 11828247

[B98] Turgut M.. (2021). Sportive university students and life satisfaction. Kibrisli Egitim Bilimleri Dergisi 16, 423–435. 10.18844/cjes.v16i1.5545

[B99] Varca P. E. Shaffer G. S. Saunders V. (2010). A longitudinal investigation of sport participation and life satisfaction. J. Sport Psychol. 6, 440–447. 10.1123/jsp.6.4.440

[B100] Wang G. H. Li W. D. Dou K. (2020). Extracurricular sports participation increases life satisfaction among Chinese adolescents: a moderated mediation model. Soc. Behav. Pers. Int. J. 48, 1–11. 10.2224/sbp.8993

[B101] Wang J. L.. (2003). A study on sports cognition,motivation and participation of different career ladies in xi'an china. J. Hubei Sports Sci. 22, 295–297.

[B102] Weiss M. R. Smith A. L. Theeboom M. (1996). “That's what friends are for”: children's and teenagers' perceptions of peer relationships in the sport domain. J. Sport Exerc. Psychol. 18, 347–379. 10.1123/jsep.18.4.347

[B103] Wheeler S.. (2012). The significance of family culture for sports participation. Int. Rev. Sociol. Sport 47, 235–252. 10.1177/1012690211403196

[B104] Wigfield A.. (1994). Expectancy-value theory of achievement motivation. Contemp. Educ. Psychol. 25, 68–81. 10.1006/ceps.1999.1015 10620382

[B105] Xiao Y. Ren X. Zhang P. Ketlhoafetse A. (2020). The effect of service quality on foreign participants' satisfaction and behavioral intention with the 2016 Shanghai International Marathon. Int. J. Sports Mark. Sponsor. 21, 91–105. 10.1108/IJSMS-04-2019-0037

[B106] Yan Y. Y. Li N. (2019). A study on the self-efficacy of sports skill formation among students majoring in physical education. Bull. Sport Sci. Technol. 27, 114–116. 10.19379/j.cnki.issn.1005-0256.2019.05.048

[B107] Zhang B. Wang Y. (2019). Research on the influence of parents' sports participation on college students' sports motivation and behavior. J. Shangrao Normal Univ. 39, 116–120.

[B108] Zhang J. X. Schwarzer R. (1995). Measuring optimistic self-beliefs: a Chinese adaptation of the general self-efficacy Scale. Psychol. Int. J. Psychol. Orient 38, 174–181.

